# Supplementary material for: Endocannabinoid System Components of the Female Mouse Reproductive Tract Are Modulated during Reproductive Aging
Source: Int J Mol Sci. 2023 Apr 19;24(8):7542. doi: 10.3390/ijms24087542 (PMC10144466; doi:10.3390/ijms24087542)
Supplement: Supplementary file 1 [file ijms-24-07542-s001.zip › ijms-2335731-supplementary.pdf]

Supplementary material

Table S1. Statistical significance of ELISA assay among the different reproductive age groups.

**P values**	**CB_1**	**CB_2**	**GPR55**	**TRPV1**	**NAPE-PLD**	**FAAH**	**DAGL-α**	**DAGL-β**	**MAGL**
**Ovaries**									
$P < 0.05$	-	PR *vs* Ad	-	PrP *vs* Ad	LR *vs* PR	PrP *vs* Ad	-	-	-
$P < 0.01$	Ad *vs* PR	LR *vs* PR	-	-	PrP *vs* Ad	PR *vs* Ad<br>Ad *vs* LR	-	Ad *vs* LR<br>PrP *vs* Ad	-
$P < 0.001$	PrP *vs* Ad<br>PrP *vs* PR<br>LR *vs* PR<br>LR *vs* Ad<br>LR *vs* PrP	LR *vs* Ad<br>LR *vs* PrP	-	PR *vs* PrP<br>PR *vs* LR<br>PR *vs* Ad	LR *vs* Ad	PR *vs* Ad<br>PR *vs* PrP	-	PR *vs* Ad<br>PR *vs* PrP<br>PR *vs* Ad	-
**Oviducts**									
$P < 0.05$	-	PR *vs* Ad	Ad *vs* LR<br>PR *vs* Ad	-	PrP *vs* PR<br>LR *vs* Ad<br>LR *vs* PR	-	-	PrP *vs* Ad	-
$P < 0.01$	LR *vs* PrP	LR *vs* Ad	-	-	PrP *vs* Ad	-	-	PR *vs* PrP	-
$P < 0.001$	LR *vs* Ad<br>LR *vs* PR<br>PrP *vs* Ad<br>PrP *vs* PR	PrP *vs* Ad<br>PrP *vs* LR<br>PrP *vs* PR	PR *vs* LR<br>PrP *vs* LR	PR *vs* PrP<br>PR *vs* Ad<br>PR *vs* LR	-	-	PrP *vs* Ad<br>PrP *vs* LR<br>PrP *vs* PR	PR *vs* Ad<br>PR *vs* LR<br>PrP *vs* Ad	PrP *vs* Ad<br>PrP *vs* LR<br>PrP *vs* PR
**Uteri**									
$P < 0.05$	-	-	PR *vs* PrP<br>PR *vs* Ad<br>PR *vs* LR	-	LR *vs* PrP<br>LR *vs* PR	PrP *vs* Ad<br>PR *vs* LR	-	-	-
$P < 0.01$	PrP *vs* PR	-	-	-	LR *vs* Ad	LR *vs* Ad<br>PR *vs* PrP	PR *vs* Ad	PrP *vs* Ad<br>LR *vs* Ad<br>PR *vs* Ad	-
$P < 0.001$	PrP *vs* LR<br>Ad *vs* PrP<br>Ad *vs* LR<br>Ad *vs* PR	PrP *vs* Ad<br>PrP *vs* LR<br>PrP *vs* PR	-	-	-	PR *vs* Ad	PrP *vs* Ad<br>LR *vs* Ad	-	-

Abbreviations: Ad, adult; CB_1, cannabinoid receptor type 1; CB_2, cannabinoid receptor type 2; DAGL-α and -β, diacylglycerol lipases α and β; FAAH, fatty acid amide hydrolase; GPR55, G-protein coupled receptor 55; LR, late-reproductive; MAGL, monoacylglycerol lipase NAPE-PLD, *N*-acylphosphatidylethanolamines-specific phospholipase D PR, post-reproductive; PrP, prepubertal; TRPV1, transient receptor potential vanilloid type 1 channel.
